# Transfer-printing of active layers to achieve high quality interfaces in sequentially deposited multilayer inverted polymer solar cells fabricated in air

**DOI:** 10.1080/14686996.2016.1221306

**Published:** 2016-09-12

**Authors:** Varun Vohra, Takuya Anzai, Shusei Inaba, William Porzio, Luisa Barba

**Affiliations:** ^a^ Department of Engineering Science, University of Electro-Communications, Chofu, Japan; ^b^ Istituto per lo Studio delle Macromolecole, CNR-ISMAC, Milano, Italy; ^c^ Istituto di Cristallografia-Sincrotrone Elettra, Basovizza, Italy

**Keywords:** Polymer solar cell, P3HT, PDMS, transfer, organic electronics, 50 Energy Materials, 209 Solar cell / Photovoltaics

## Abstract

Polymer solar cells (PSCs) are greatly influenced by both the vertical concentration gradient in the active layer and the quality of the various interfaces. To achieve vertical concentration gradients in inverted PSCs, a sequential deposition approach is necessary. However, a direct approach to sequential deposition by spin-coating results in partial dissolution of the underlying layers which decreases the control over the process and results in not well-defined interfaces. Here, we demonstrate that by using a transfer-printing process based on polydimethylsiloxane (PDMS) stamps we can obtain increased control over the thickness of the various layers while at the same time increasing the quality of the interfaces and the overall concentration gradient within the active layer of PSCs prepared in air. To optimize the process and understand the influence of various interlayers, our approach is based on surface free energy, spreading parameters and work of adhesion calculations. The key parameter presented here is the insertion of high quality hole transporting and electron transporting layers, respectively above and underneath the active layer of the inverted structure PSC which not only facilitates the transfer process but also induces the adequate vertical concentration gradient in the device to facilitate charge extraction. The resulting non-encapsulated devices (active layer prepared in air) demonstrate over 40% increase in power conversion efficiency with respect to the reference spin-coated inverted PSCs.

## Graphical abstract

**Figure:**
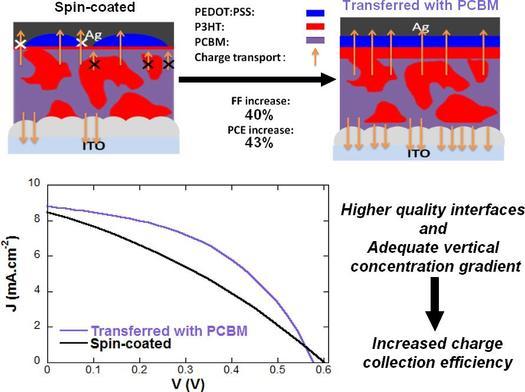

## 1. Introduction

Polymer solar cells (PSCs) [1–5] have been developed over the past decade and now reach power conversion efficiencies (PCE) overcoming the milestone value of 10%.[6–12] In order to become a realistic alternative to the state-of-the-art silicon solar cells, developers of PSCs are required to either remarkably increase their photovoltaic performances or to considerably reduce the cost of production.[13] To realistically reduce the production cost by using high-productivity technologies such as the roll-to-roll process, finding the optimized conditions and device architectures for device fabrication in air is essential to maintain relatively high photovoltaic performances. However, the fact that the state-of-the-art materials used for PSC active layers tend to degrade when exposed to oxygen and moisture remains one of the major limitations for device fabrication in air.[14–16]

The importance of the vertical distribution of electron-donor and electron-acceptor materials in the active layer of PSCs has been demonstrated through a variety of recent studies.[17–20] In fact, one of the most efficient methods to increase their PCE is to include electron-donor or electron-acceptor only buffer layers between the active layer and the anode or cathode, respectively. Some solution-processed sequential layer deposition processes have been previously introduced and resulted in successfully achieving the adequate morphologies for high efficiency solar cells.[19–25] These include using orthogonal solvents to form diffusive bilayer active layers,[21–25] using a sacrificial orthogonal solvent layer for successful spin-coating without dissolution of the underlying layer [20] and simply spin-coating the second layer on top of the underlying layer which is partially dissolved during the process.[19] One common feature of these methods is the lack of reproducibility of the results as it becomes extremely difficult in practice to control the amount of material from the first layer dissolved during the second layer deposition, especially for inverted device architectures.

Thin film transfer-printing from flexible polydimethylsiloxane (PDMS) stamps is a well-known dry transfer process for solar cell and light-emitting device micro-fabrication.[26–32] In fact, through this solvent-free deposition technique, one can sequentially deposit multilayered PSC active layers (e.g. to induce a concentration gradient in device active layers) without generating any damage to underlying layers.[26, 27] However, one of the main issues when it comes to applying this method to PSCs fabrication consists in the deposition of high quality active layers on the PDMS stamps. The poly(3-hexylthiophene-2,5-diyl): phenyl-C_61_-butyric acid methyl ester (P3HT:PCBM) materials in chlorinated organic solvents cannot properly wet the PDMS surface and thus require PDMS surface energy modification. In a previous transfer-printing process for PSCs, such a surface modification was obtained by spin-coating chloroform prior to deposition of the P3HT:PCBM solution in chloroform.[26] As chloroform is a volatile solvent which easily diffuses inside PDMS, this method resulted in the formation of thin spin-coated layers of P3HT:PCBM on PDMS. However, due to the PDMS swelling induced by chloroform diffusion and chloroform’s volatility, it may be difficult to deposit high quality films in a reproducible manner.

Here, we propose an alternative method where PEDOT:PSS is first deposited from an aqueous suspension on a plasma treated PDMS surface, followed by spin-coating of the P3HT:PCBM solution. The PEDOT:PSS/P3HT:PCBM bilayers are then transferred from the PDMS stamps to zinc oxide (ZnO) covered indium tin oxide (ITO) with or without the presence of a PCBM interlayer with all the process done in air. Our study includes calculations of works of adhesion to predict whether PEDOT:PSS/P3HT:PCBM bilayers can be successfully transferred from PDMS to the underlying substrates. The calculations are correlated with experimental results demonstrating that ideal transfer conditions are obtained when printing PDMS/PEDOT:PSS/P3HT:PCBM on PCBM/ZnO/ITO substrates. As PEDOT:PSS and PCBM can act as hole only and electron only layers respectively in an inverted PSC architecture, the results presented here demonstrate three key surface and interface aspects to successfully fabricate inverted PSCs with a vertical concentration gradient. (1) The insertion of PEDOT:PSS facilitates the deposition of P3HT:PCBM solutions on PDMS. (2) PEDOT:PSS/P3HT:PCBM layers’ transfer is facilitated with a PCBM interlayer inserted on top of the ZnO substrate. (3) The optimized conditions for transfer-printing also correspond to the ideal device architectures in inverted PSCs. Furthermore, this method not only results in the fabrication of sequentially deposited multilayer PSC active layers but also provides means to fabricate a homogeneous PEDOT:PSS layer between the active layer and the metal electrode in inverted PSCs. The devices prepared using the transfer process exhibit an average increase of 40% with respect to the reference spin-coated cells, demonstrating that PCE up to 2.4% can be obtained with P3HT:PCBM active layers processed entirely in air.

## 2. Experimental section

### 2.1. Materials and thin film preparation

PDMS elastomer (Sylgard 184) was purchased from Dow Corning (Midland, MI, USA). For the fabrication of stamps, the PDMS precursor was mixed with the curing agent (10:1 weight ratio) and stirred using a glass micropipette. After undergoing a standard cleaning procedure (in acetone, surfactant, deionized water, isopropanol and hot isopropanol), standard microscope glass slides with polished surface were used as substrate for the mixture deposition and stamp fabrication. After removing the air bubbles formed during stirring, the mixture was deposited on the cleaned glass substrates and cured at 80 °C for at least two hours. PDMS stamps were treated for 30 min in a plasma generated using a pressure of 200–600 mTorr with air and a light intensity of approximately 10 W (at 10 MHz).

The ZnO layers were prepared on either plasma treated cleaned glass or ITO covered glass substrates. A precursor mixture composed of 500 mg ZnOAc·H_2_O and 101.4 mg 2-aminoethanol in 5 ml of 2-methoxyethanol was stirred for 3 h at room temperature. Once a homogenous solution was obtained, the mixture was spin-coated on the substrates at a speed of 3000 rpm for 30 s. The resulting layers were annealed at 200 °C for 30 min followed by slow cooling to room temperature.

All solvents were acquired from Sigma-Aldrich (St Louis, MO, USA). PEDOT:PSS (AI4083), P3HT and PCBM were purchased from Heraeus-Clevios (Hanau, Germany), Rieke Metals (Lincoln, NE, USA) and Luminescent Technology (Hsin-Chu, Taiwan), respectively. For the P3HT:PCBM blends in chlorobenzene (CB), the weight ratio of P3HT:PCBM was maintained at 1:0.8 with a total concentration of 45 mg ml^–1^ and the spin-coating speed was optimized to obtain approximately 150 nm thick active layers. The PCBM underlayer was spin-coated from a 10 mg ml^–1^ solution at a speed of 1000 rpm for 30s. Triton-X (purchased from Sigma-Aldrich) was added as a wetting agent to the PEDOT:PSS suspension (1 vol.%) and the PEDOT:PSS thin films were spin-coated at 1600 rpm for 60 s on either PDMS or the P3HT:PCBM layer. In the latter case, we ensured that the P3HT:PCBM layer was fully dried by keeping the substrates in a vacuum chamber for at least 30 min.

### 2.2. Device preparation and characterization

For the device fabrication, the cleaning and spin-coating conditions were as described above. The non-encapsulated devices were finalized by evaporating a 100 nm thick silver layer acting as a reflective electrode and characterized using a sourcemeter (Keithley 2401, Keithley Instruments, Solon, OH, USA) and a solar simulator (SAN-EI Electric, Osaka, Japan, AM 1.5G, 100 mW cm^–2^) at room temperature to evaluate their photovoltaic performances. The spin-coated devices (reference) were annealed at 150 °C for 10 min which corresponds not only to the optimized annealing time for spin-coated P3HT:PCBM devices with similar device architectures but also to the optimized transfer conditions in the case of transfer-printing (as discussed below). The printing process was carried out using a glass plate and weights deposited on top of the PDMS stamps with an optimized printing pressure of 400 Pa. The process was performed on 10 substrates for each condition (e.g. with and without the insertion of a PCBM underlayer) to estimate the transfer ratios and their standard deviations.

### 2.3. Morphological characterizations

Contact angles were measured by dropping 50 μl of test solvent onto the characterized surfaces and taking a photograph from a horizontally aligned position. The precise angles were measured using ImageJ with a plugin developed and provided by the Biomedical Imaging Group of the Ecole Polytechnique Federale de Lausanne.[33] The thicknesses and surface morphologies of the prepared thin films were characterized by a Dektak profilometer (Bruker, Billerica, MA, USA) and atomic force microscope (AFM) respectively. Grazing-incident X-ray diffraction (GI-XRD) measurements were performed at the X-ray diffraction beamline 5.2 at the Synchrotron Radiation Facility Elettra in Trieste (Italy). Two-dimensional diffraction patterns were recorded with a 2 M Pilatus silicon pixel X-ray detector (DECTRIS Ltd, Baden, Switzerland). The sample inclination to the beam was changed from *ω* = 0.03° to *ω* = 0.2° to characterize the surface (5 nm depth) and bulk properties of the samples, respectively. Detailed measurement conditions and data treatment can be found in a previously published work.[34]

## 3. Results and discussion

### 3.1. Dependence of film quality on PDMS wettability

The first step of our work consisted in efficiently depositing P3HT:PCBM layers on the PDMS stamps. To obtain homogeneous P3HT:PCBM films on PDMS, we investigated two potential deposition methods described below:

• Method A: direct deposition of the P3HT:PCBM solution in CB on plasma treated PDMS.
• Method B: deposition of PEDOT:PSS + surfactant on plasma treated PDMS, followed by deposition of P3HT:PCBM solution from CB.

To predict the behavior of these two methods, following the methodology introduced by Young-Dupré for liquid–surface interactions, we calculated the spreading parameters (S_SL_) between the solvent (water or water + surfactant for PEDOT:PSS and CB for P3HT:PCBM solutions, respectively) and PDMS before and after plasma treatment.[35–37] The calculations for S_SL_ are based on contact angle measurements between the various solvents and PDMS substrates and follow Equation (1).[35,36]

(1) $${\text{S}}_{\text{SL}}=\gamma_{\text{L}}\left(\cos\theta -1\right)$$

where γ_L_ is the surface tension of the liquid L and *θ* the contact angle measured when depositing a drop of liquid L on the surface S. It should be pointed out that the spreading capacity of the liquid on the surface becomes higher when the values of S_SL_ get closer to zero (S_SL_ ≥ 0 corresponding to complete spreading of the liquid over the surface). Furthermore, the substrate coverage and film quality is directly dependent on the spreading of the solution on the substrate.

**Figure 1.**
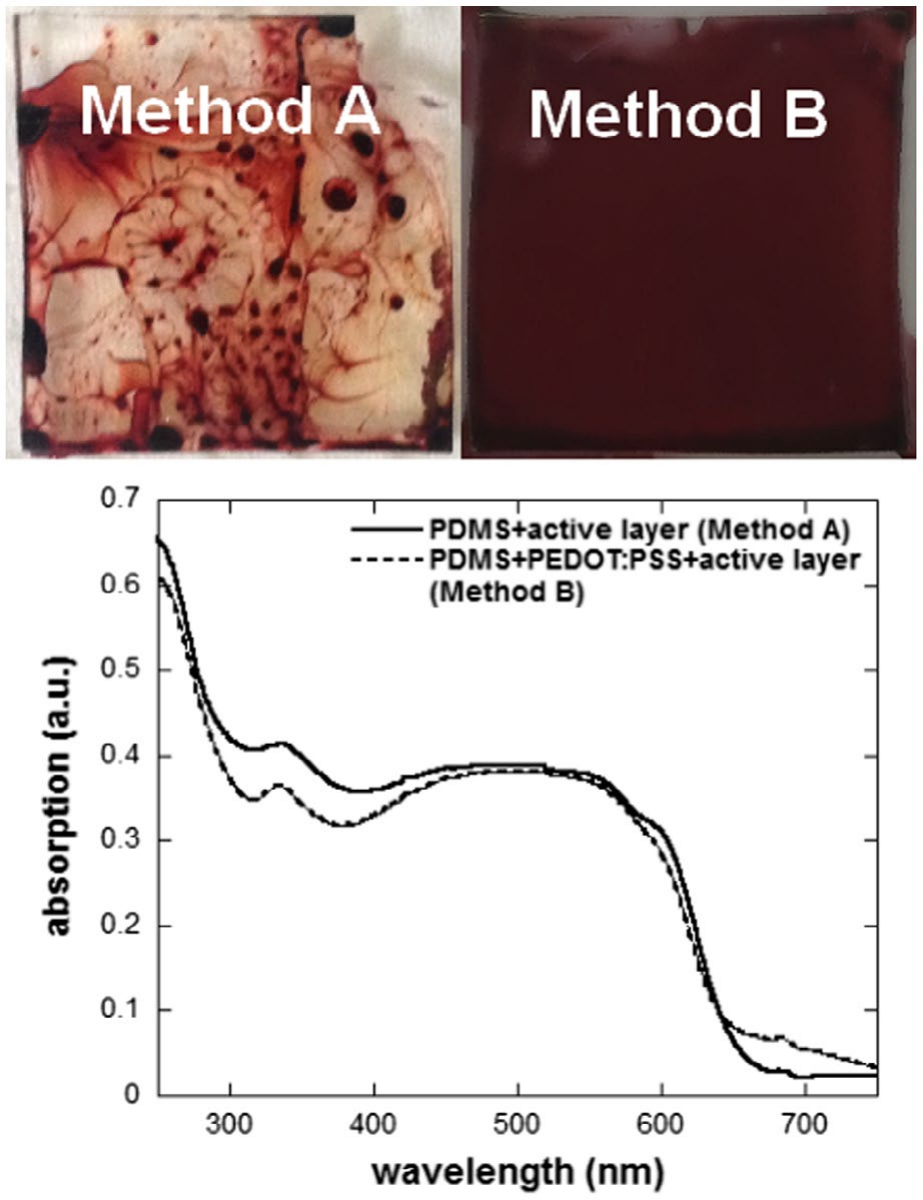
Photographs (top) and absorption spectra (bottom) of P3HT:PCBM films deposited following method A (directly on PDMS stamps) and method B (on PEDOT:PSS covered PDMS stamps). The PDMS substrate size is 20 × 20 mm^2^.

The resulting values for S_SL_, together with the γ_L_ and *θ* values for the test solvents, are summarized in Table 1. These calculations indicate that, once PDMS undergoes a 30 min surface plasma treatment, water containing the surfactant will spread more easily on its surface as compared to CB or water without the surfactant. Note that, unlike water, we observed that CB swells PDMS as CB molecules diffuse inside the PDMS network. Similarly to previous studies in which chloroform was spin-coated on the PDMS surface to modify its surface energy,[26] the CB trapped at the surface of the PDMS stamp acts as a compatibilizing agent between PDMS and the CB drop on its surface and consequently, S_SL_ for CB might be slightly overestimated. Moreover, the diffusion of CB inside PDMS induces changes in P3HT:PCBM solution concentration (increase in actual spin-coated solution concentration) and the poor solubility of P3HT may lead to the formation of more crystalline domains during the direct P3HT:PCBM deposition on PDMS. The change in P3HT crystallinity due to CB diffusion inside the PDMS stamp is confirmed by the absorption spectra of the two films in Figure 1. The absorption spectrum of the P3HT:PCBM film deposited directly on PDMS (method A) exhibits more defined absorption shoulders around 580 and 610 nm, which are commonly ascribed to a higher degree of P3HT crystallinity. Note that, depending on the area measured on the samples prepared using method A, the absorption intensity varies (changes in film thickness) but the spectral shape remains the same. Figure 1 displays photographs of the P3HT:PCBM films obtained using methods A and B. Unlike method A, a very homogeneous film (both in color and aspect) can be obtained in the case of method B. These results clearly demonstrate that the insertion of the PEDOT:PSS layer allows to deposit much higher quality P3HT:PCBM films.

**Table 1.** Predicted wetting on PDMS before and after plasma treatment.

PDMS	Before plasma treatment			30 min plasma treatment		
	CB	H_2_O	H_2_O Triton	CB	H_2_O	H_2_O Triton
γ_L_ (mJ m^−2^)	33.6	72.8	33.0*	33.6	72.8	33.0*
*θ* (°)	50.9	75.8	71.2	27.9	12.0	11.6
S_SL_ (mJ m^−2^)	−12.4	−54.9	−22.4	−3.91	−1.59	−0.67

* data provided by the manufacturer for a 1 vol.% concentration in water.

### 3.2. Theoretical calculations and experimental results of the transfer-printing process

To assess whether the printing process can be successfully realized using the PDMS/PEDOT:PSS/P3HT:PCBM sequentially deposited layers, we calculated the works of adhesion between the various layers involved (Equation (2)) using surface free energy values found in literature to predict which transfers are more probable.[35,36]

(2) $${\text{W}}_{\text{S1/S2}}\;=2\;\left(\sqrt{\gamma_{\text{S}1}^{\text{d}}}\sqrt{\gamma_{\text{S}2}^{\text{d}}}+\sqrt{\gamma_{\text{S}1}^{\text{p}}}\sqrt{\gamma_{\text{S}2}^{\text{p}}}\right)$$

where γ_S_ is the surface free energy of solid S and d and p correspond to its dispersive and polar components, respectively.

A higher value of W_s1/s2_ corresponds to a higher degree of adhesion between two surfaces.[35–37] The results presented in Table 2 can therefore be used to predict both the quality of the interface and whether the transfer-printing process will be successful or not. For instance, W_PEDOT:PSS/P3HT:PCBM_ is found to have a higher value compared to W_PDMS/PEDOT:PSS_, as well as W_P3HT:PCBM/ZnO_, and W_P3HT:PCBM/PCBM_. Therefore, if the printing process is successful, both P3HT:PCBM and PEDOT:PSS layers are expected to be transferred together.

**Table 2.** Works of adhesion at the various interfaces involved in the printing process.

Ws_1_/s_2_ (γ_/_γ^d^_/_γ^p^) [mJ m^−2^]	PDMS	PEDOT:PSS	P3HT:PCBM	ZnO	PCBM
PDMS (19.9/17.9/2.0)[38]	–	70.6	48.9	–	–
PEDOT:PSS (73/42/31)[39]	70.6	–	74.9	–	–
P3HT:PCBM (33.4/33.4/0)[40]	48.9	74.9	–	54.2	72.9
ZnO (40.5/22/18.5)[41]	–	–	54.2	–	86.1
PCBM (50.6/39.8/9.8)[42]	–	–	72.9	86.1	–

**Figure 2.**
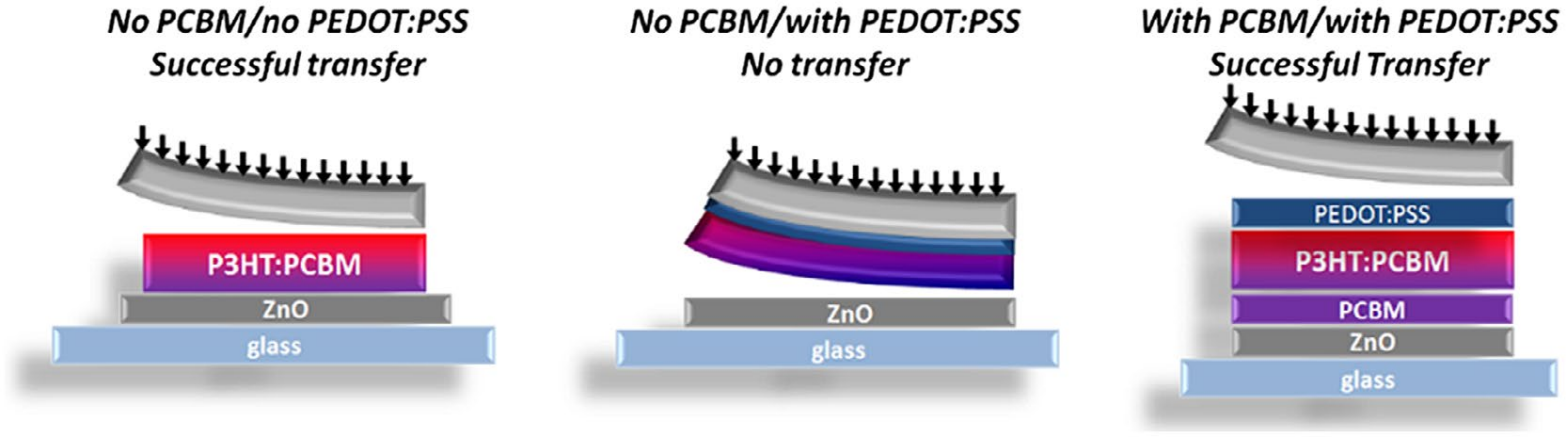
Schematic representations of the predicted results for the transfer-printing process with and without PEDOT:PSS and PCBM interlayers.

On the other hand, W_PDMS/PEDOT:PSS_ has a higher value compared to W_P3HT:PCBM/ZnO_ while W_PDMS/P3HT:PCBM_ is just slightly lower than W_P3HT:PCBM/ZnO_. Through these calculations, we predict that P3HT:PCBM (with no underlying PEDOT:PSS interlayer) on PDMS should be successfully transferred from PDMS to ZnO. However, the PEDOT:PSS/P3HT:PCBM should, according to these calculations, stay on the PDMS side during the printing process. Last but not least, the deposition of a PCBM thin layer on top of the ZnO substrate acts as a compatibilizing layer as it strongly bonds to ZnO and should allow for an easier printing process of the PEDOT:PSS/P3HT:PCBM bilayer from PDMS to the ZnO/PCBM substrate. The predicted results of the various potential transfer-printing attempts are summarized in Figure 2.

**Figure 3.**
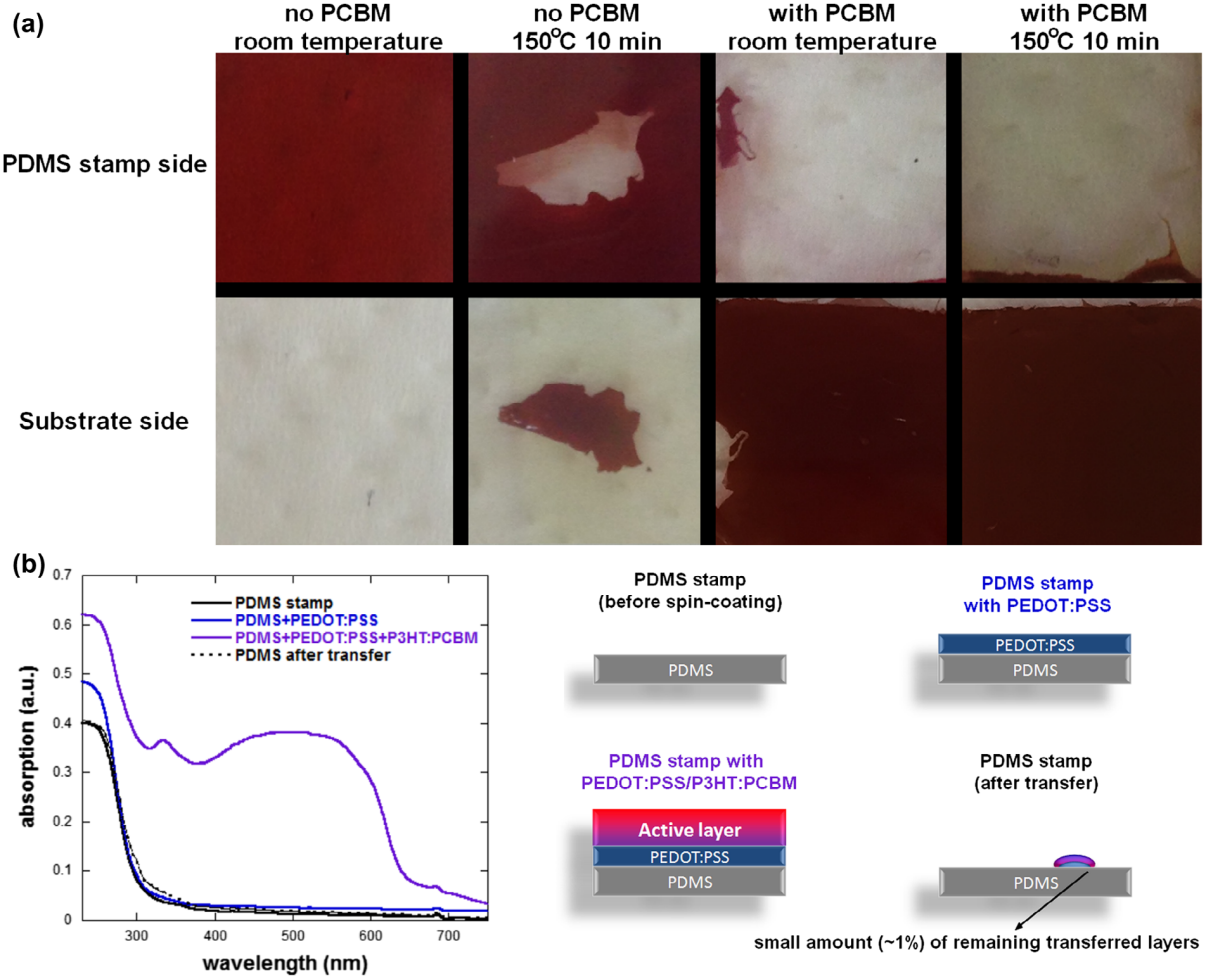
(a) Typical transfer results of PEDOT:PSS/P3HT:PCBM films printed on ZnO substrates without and with the presence of a PCBM interlayer. (b) Absorption spectra of PDMS stamps at various stages of the deposition and transfer process. The PDMS substrate size is 20 × 20 cm^2^.

As a high quality P3HT:PCBM layer cannot be obtained directly on PDMS in a controllable way, we focused on the two situations with PEDOT:PSS and with or without PCBM. For both types of experiment, the printing process was optimized in terms of applied pressure, temperature and time. Figure 3 displays the typical transfer results for an applied pressure of 400 Pa for 10 min either at room temperature or at 150 °C. It is worth noticing that longer printing process times gave similar results. However, as in P3HT:PCBM PSCs, the optimized annealing temperature and time are also 150 °C for 10 min, so all samples prepared for device fabrication were obtained following the same protocol. The theoretical calculations predict that no transfer should be obtained when printing PDMS/PEDOT:PSS/P3HT:PCBM layers on ZnO substrates. On the other hand, PEDOT:PSS/P3HT:PCBM layers should be transferred from the PDMS stamps to PCBM covered ZnO substrates. This is well reflected in the results presented in Figure 3 when the transfer process is conducted at room temperature. However, by increasing the temperature to 150 °C, we were able to partially transfer the P3HT:PCBM layer to the ZnO substrates with an average transfer ratio of approximately 26% for 20 × 20 mm^2^ samples. Note that the standard deviation of these transfers is extremely large (±38%) as the transfer process in the case of the ZnO substrates with no PCBM has a low reproducibility and generally results in either one of the following situations: low transfer (approx. 1% transfer ratio), partial transfer (approx. 25% transfer ratio) or full transfer (over 90% transfer ratio). The variation between the transfer ratios may be due to lateral strain during the positioning and lift-off of the PDMS stamp. Furthermore, similarly to previously published results, we found that our preparation method results in the formation of nanoripples on the surface of the deposited ZnO thin films.[20] Due to these nanoripples, the actual contact area between the P3HT:PCBM film to be transferred (flat) and the ZnO substrate (rippled) varies from one test to another and may be one of the reasons for the low reproducibility of the results. Figure 3 clearly demonstrates that when a thin PCBM layer is deposited on the ZnO substrates, a very high quality transfer can be obtained resulting in smooth and homogeneous P3HT:PCBM/PEDOT:PSS layers both at room temperature and at 150 °C. The transfer ratios measured at room temperature and 150 °C are 96 ± 3% and 98 ± 1%, respectively. To verify that the PEDOT:PSS layer is successfully transferred together with the active layer, we performed UV-vis absorption measurements of the PDMS stamps at the various stages of the process for samples with high transfer ratios: PDMS substrates; after deposition of the PEDOT:PSS layer; after deposition of the P3HT:PCBM layer; after transferring the PEDOT:PSS/P3HT:PCBM layers to the substrate. The measurements were performed on the same PDMS substrates at the various stages to avoid any changes in absorption spectra due to variations in PDMS thickness. Although PEDOT:PSS and PDMS have an overlapping absorption spectral range, we can clearly observe that the transferred PDMS substrate has a very similar absorption spectrum to that of the PDMS substrate prior to PEDOT:PSS/P3HT:PCBM layers deposition. The small differences between the two absorption spectra can be assigned to the small amount of PEDOT:PSS/P3HT:PCBM (less than 1% for the measured samples) which was not transferred to the substrate during the process. The absorption spectra clearly demonstrate that the strong adhesion between the PEDOT:PSS and the P3HT:PCBM layers results in both layers being transferred at the same time during the process.

### 3.3. Application to photovoltaic device fabrication

**Figure 4.**
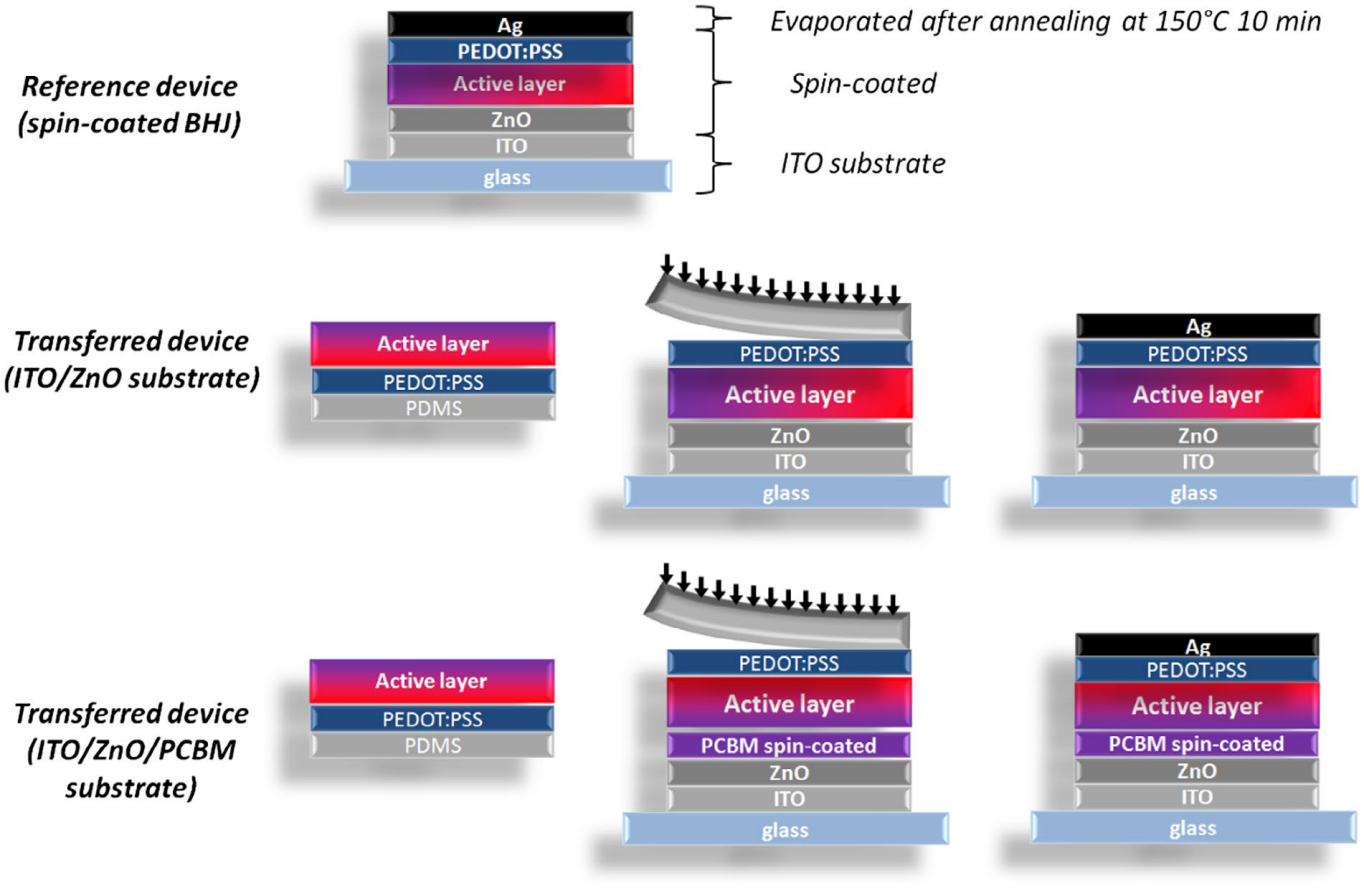
Schematic representations of the three types of devices prepared to compare the performances of devices fabricated by the transfer-printing process (transferred devices) with the traditional process (reference spin-coated device).

Figure 4 summarizes the three types of devices that were prepared in this study. In the reference device, the active layer (P3HT:PCBM) is spin-coated directly on the glass/ITO/ZnO substrates which act as the cathode. A hole conducting PEDOT:PSS layer is then spin-coated on top of the active layer and the device is finalized by evaporating a 100 nm thick silver (Ag) anode. The two investigated device types correspond to the transferred devices (PEDOT:PSS/active layer) with and without the insertion of the PCBM interlayer on top of the glass/ITO/ZnO substrates.

The addition of surfactants to a PEDOT:PSS dispersion has been previously reported as an efficient method to spin-coat PEDOT:PSS layers on top of PSC active layers.[43,44] However, in our case, this method did not result in the formation of high quality PEDOT:PSS layers (inhomogeneity could be observed by eye). To further emphasize the relevance of the transfer-printing process presented in this work, we employed a contact angle approach to estimate the compatibility of the PEDOT:PSS + surfactant suspension with either plasma treated PDMS or P3HT:PCBM active layer’s surfaces. Although we could not estimate γ_L_ for the suspension, as the solution remains the same for both experiments, we can still quantitatively compare the S_SL_ on the two surfaces.

**Figure 5.**
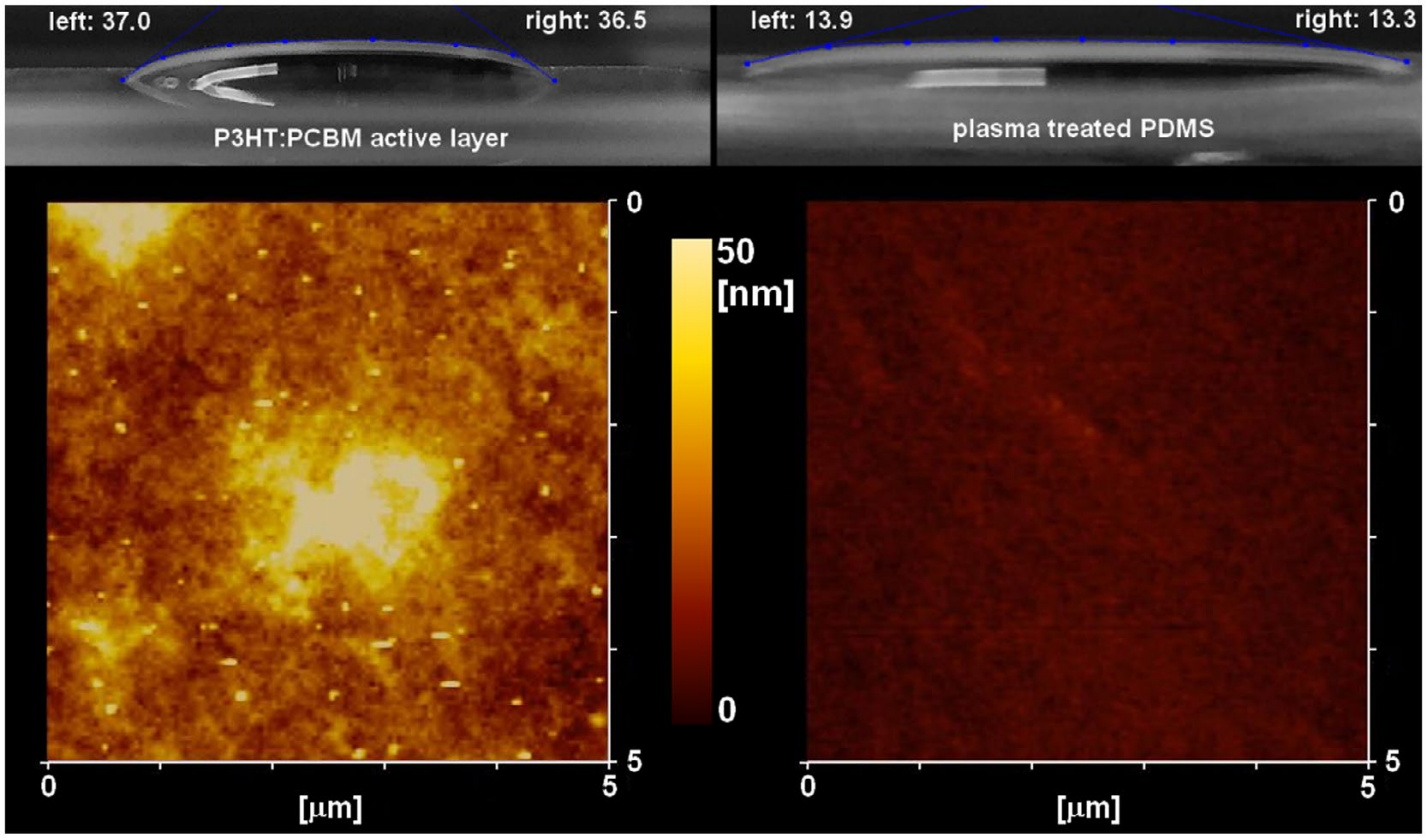
Contact angle measurements of the PEDOT:PSS + surfactant solution used for device fabrication on P3HT:PCBM active layer and plasma treated PDMS surfaces. The AFM images correspond to the PEDOT:PSS surfaces deposited on each layer.

From the contact angle measurements in Figure 5, we can extrapolate the values of cos*θ*−1 which are used for S_SL_ calculations (Equation (1)).[35,36] The cos*θ* values for PDMS and P3HT:PCBM active layers are 0.97 and 0.80, respectively. As γ_PEDOT:PSS+surfactant_ is a constant, we can calculate the ratio S_PDMS/PEDOT:PSS+surfactant_/S_P3HT:PCBM/PEDOT:PSS+surfactant_ which, accordingly to the measured contact angles, is equal to 0.15. Note that a smaller absolute value of S_SL_ corresponds to a better spreading of the solution on the surface for contact angles with values up to 90° and we can therefore conclude that the suspension spreads much better on PDMS than on the P3HT:PCBM active layers (visually confirmed in Figure 5). From the AFM measurements displayed in Figure 5, we extrapolated the root mean square surface roughness (RMS) and maximum height (Ry) for the PEDOT:PSS layers formed by direct spin-coating on P3HT:PCBM films or transfer-printing. The RMS values were 9.89 and 2.09 nm, respectively, with Ry values reaching 48.36 and 6.58 nm, respectively. This confirms our observation that better quality PEDOT:PSS layers are formed during the printing process with respect to the direct spin-coating of PEDOT:PSS on the active layers. Furthermore, the large surface roughness observed for PEDOT:PSS spin-coating on P3HT:PCBM films may lead to an increase of the series resistance (Rs) at the PEDOT:PSS/metal anode interface.

### 3.4. Effect of the fabrication process on the device performances

**Figure 6.**
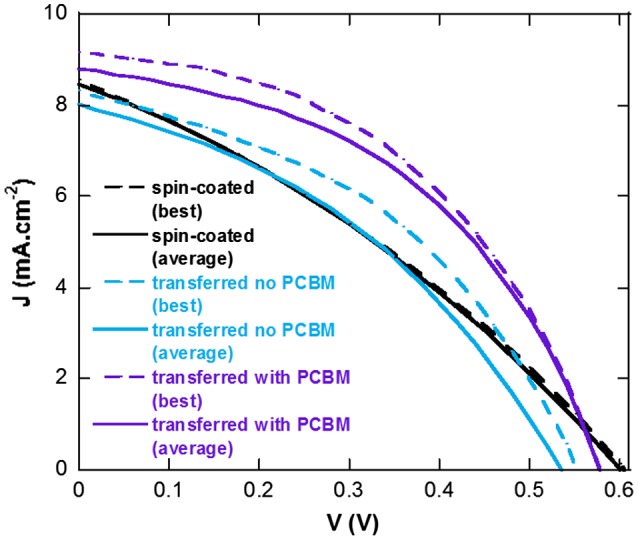
Average (solid line) and best (dashed line) J-V characteristics of spin-coated and transferred devices with and without the PCBM underlayer.

The device parameters measured from the three types of devices are summarized in Table 3 and their J-V characteristics are displayed in Figure 6. In both Table 3 and Figure 6, we present the average performances and standard deviation of PCE for eight devices as well as the best performing device for each device type. From the J-V curves, we can clearly observe that a higher FF is obtained when using the transfer-printing process as compared to spin-coating which is partially due to the formation of a higher quality PEDOT:PSS layer (lower Rs) and a complete coverage at its interface with P3HT:PCBM in the case of transfer-printing. Hole collection is highly affected by the quality of the PEDOT:PSS layer which explains the large changes observed in Rs and FF between the spin-coated and printed devices.

**Table 3.** Photovoltaic parameters extracted from the J-V curves for the studied devices.

	Jsc (mA cm^−2^)	Voc (V)	FF (%)	PCE (%)	Rs (Ω cm^2^)	Rsh (Ω cm^2^)
Spin-coated (average)	8.36	0.60	32.2	1.64 ± 0.02	42.3	133.7
Spin-coated (best)	8.57	0.60	32.0	1.65	45.9	128.3
Transferred no PCBM (average)	7.94	0.55	36.9	1.62 ± 0.23	30.5	178.1
Transferred no PCBM (best)	8.35	0.57	40.6	1.92	24.3	179.1
Transferred with PCBM (average)	8.79	0.59	45.2	2.34 ± 0.09	19.4	311.5
Transferred with PCBM (best)	9.17	0.59	45.7	2.45	18.6	464.0

However, as a large difference in FF can be observed between transferred devices without and with PCBM, we can assume that the donor-acceptor vertical concentration gradient in the active layers of our devices also greatly influences the FF. In fact, active layers spin-coated from CB have a tendency to generate such vertical concentration gradients which can be influenced by both the materials at the interfaces and the annealing conditions.[45] The shunt resistances (Rsh) values, which reflect the quantity of leakage current in the devices, suggest that a more adequate concentration gradient (higher Rsh values) is obtained for transferred devices (with or without PCBM) as compared to the reference spin-coated devices. However, the lower quality of PEDOT:PSS layer in the reference devices may also affect Rsh values (inhomogeneous hole collection layer). In fact, in our laboratory, devices with spin-coated active layers prepared in air and evaporated molybdenum trioxide as hole collection layers display average FF and PCE of 43.1 and 1.99%, respectively.

**Figure 7.**
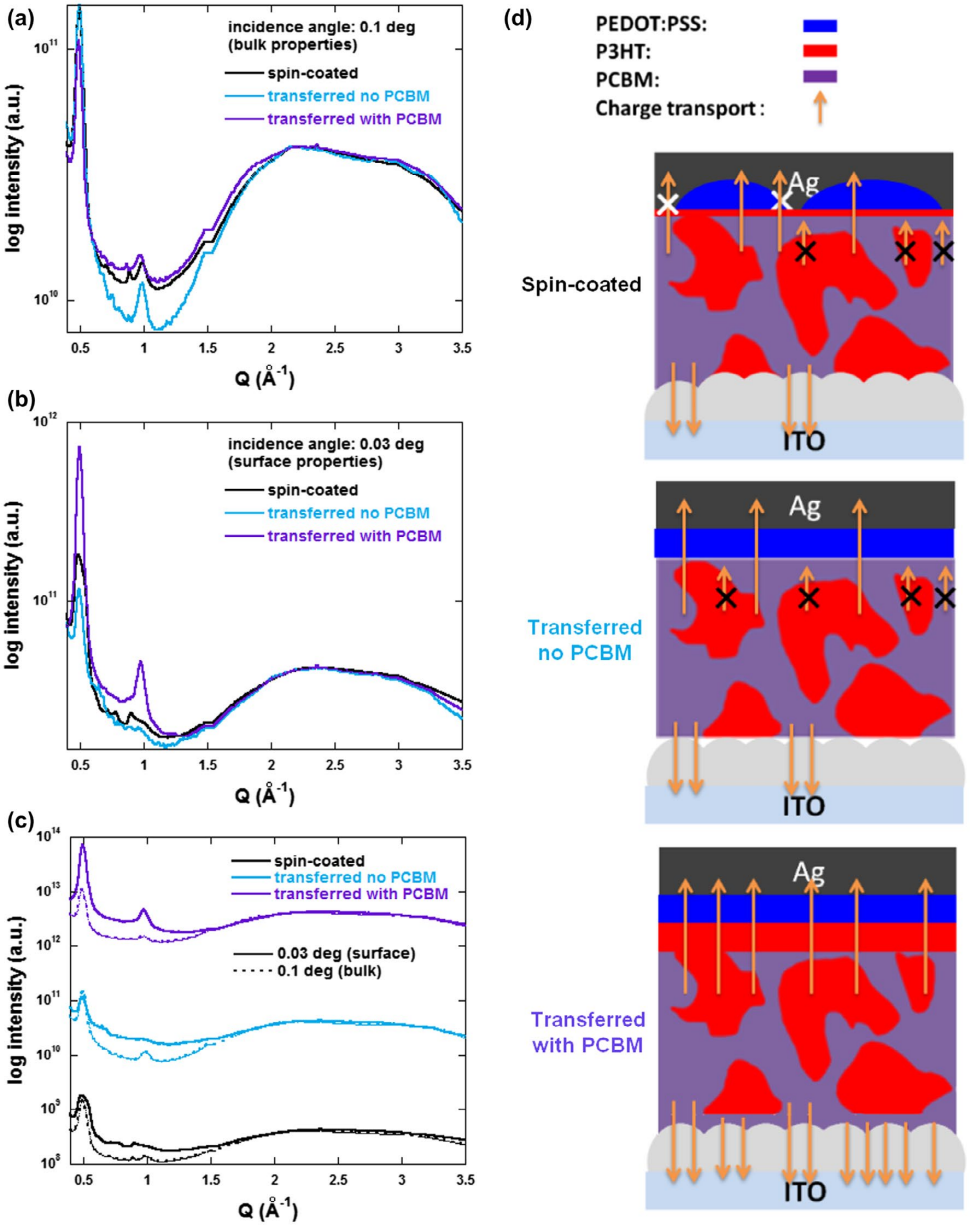
(a) Incident (0.1 degree), (b) grazing (0.03 degree) and (c) comparative XRD out-of-plane profiles extracted from 2D images of spin-coated and transferred devices. (d) Schematic representations of the active layer morphologies and charge transport in the various devices.

To observe whether vertical concentration gradients are formed in the active layers of our spin-coated and transfer-printed devices, we performed GI-XRD measurements to characterize their surface and bulk properties independently. PEDOT:PSS layers were fully removed using water prior to measurement. Figure 7 displays the spectra obtained at grazing (surface properties) and incident (bulk properties) angles for the three types of samples. The peak observed at a Q value of approximately 0.5 Å^−1^ is attributed to the (100) reflection of P3HT crystal while the diffuse peak with a maximum at approximately 2.2 Å^−1^ can be ascribed to PCBM. To compare the P3HT and PCBM ratios in a systematical manner, all the spectra were normalized to the PCBM peak maximum. In Figure 7(a) (bulk properties), we can clearly observe that the sequentially deposited active layer (PCBM/P3HT:PCBM) exhibits a lower concentration in P3HT as compared to the other two samples. This confirms that the incident angle induces enough penetration depth to characterize the bulk properties of the whole active layer (underlying PCBM layer included).

Figure 7(b) and 7(c) reveal the relative P3HT:PCBM concentration at the surface of the samples. We can observe that, compared to the spin-coated reference samples, a smaller amount of P3HT content can be measured in the case of transfer-printed films with no underlying PCBM but with the insertion of the PCBM underlayer between the transferred layer and the ZnO substrate, we induced the formation of a much higher P3HT concentration layer at the top interface of the active layer. Consequently, the favorable vertical concentration gradient generated in the devices printed on ITO/ZnO/PCBM results in the decrease of Rs and increase of Rsh, when compared to the devices printed on ITO/ZnO substrates. This leads to FF values of 36.9 and 45.2% for the transferred devices without and with PCBM, respectively which corresponds to a 22.5% increase in FF upon insertion of the PCBM underlayer for the transfer process. Note that the highest FF obtained for the transferred devices without PCBM is of approximately 40%.

Figure 7(d) schematically shows the evolution of the device morphology and its impact on the charge transport properties. A larger number of arrows corresponds to a more efficient charge collection. The charge collection efficiency not only influences the FF of the devices but also, in a much more moderate manner, their Jsc. In fact, the average Jsc values for the transferred devices only display a ± 5% variation with respect to the reference devices. On the other hand, the lower value of Voc obtained for the printed devices on ZnO directly, namely 0.55 V, did not meet our expectations. This may be due to the lower quality of ZnO/P3HT:PCBM interfaces in these devices which affects the energetic levels of the molecules present at this interface.[20] As mentioned previously, the ZnO layers, prepared using the procedure described in the experimental section, display some nanoripple formation resulting in a surface roughness (Ry) value of approximately 32.5 nm.[20] The deposition of the PCBM layer is therefore not only essential to induce a more adequate vertical concentration gradient but also to smoothen the P3HT:PCBM/ZnO interface which also, as demonstrated above, clearly enhances the facility of the transfer-printing process. Consequently, the overall PCE increases from 1.64% for spin-coated devices to 2.34% for printed devices with the PCBM interlayer. Note that, as mentioned above, all devices’ active layers were prepared in air and were characterized without encapsulation (exposed to oxygen and moisture). The oxygen and water molecules which diffused inside the active layer may act as trap sites which explains the lower FF (and PCE) observed in our work as compared to other works on P3HT:PCBM inverted PSCs.[15,44–47] However, our printed device performances (PCE reaching 2.34%) overcomes the PCE of similar device architectures processed in air (PCE of 1.79%), confirming its potential for applications to roll-to-roll compatible process in air.[15] Furthermore, the influence of the interlayers and the formation of a vertical concentration gradient can be clearly observed as inserting the PCBM interlayer and using the transfer-printing process results in a 40% increase of the device PCE compared to the reference spin-coated devices. In fact, due to the formation of an inverted concentration gradient, the printed devices (FF and PCE of 45.2 and 2.34%, respectively) even overcome the performances of devices spin-coated in air with a vacuum evaporated molybdenum trioxide hole collecting layer (FF and PCE of 43.1 and 1.99%, respectively).

## 4. Conclusions

In summary, using theoretical calculations correlated with practical experiments, we have introduced an efficient method to transfer PEDOT:PSS/active layers from PDMS stamps to the underlying substrates. Our approach, based on calculations of spreading parameters and works of adhesion between the different solutions and surfaces, not only explains the results observed in the practical experiments but also represents a powerful tool which could be applied to other transfer or lamination processes. Furthermore, we presented a method to successfully deposit high quality P3HT:PCBM films on PDMS through the insertion of a PEDOT:PSS interlayer. Although, from a theoretical point of view, the PEDOT:PSS interlayer reduces the chances for transfer from PDMS to ZnO substrates, we have demonstrated that depositing a PCBM layer prior to printing highly increases the rate of transfer from PDMS to the substrate. This alternative process not only allows for the formation of sequentially deposited layers in a controlled manner but also induces changes in vertical concentration gradients in the active layer of inverted architecture PSCs. The printed devices with the PCBM interlayer exhibit a 40% increase in PCE when compared with the spin-coated devices. This increase can be ascribed not only to the more adequate vertical concentration gradient in the printed devices but also to a higher quality PEDOT:PSS layer. Through our study, we emphasize the importance of interface quality and interactions in organic electronics. More specifically, we demonstrated that in order to successfully transfer or laminate layers, using works of adhesion can be extremely useful as it can avoid having to perform unnecessary experiments.

## Disclosure statement

No potential conflict of interest was reported by the authors.

## Funding

This work was supported by Japan Society for the Promotion of Science [grant number 26889029] and JGC-S scholarship foundation.

## References

[CIT0001] Günes S, Neugebauer H, Sariciftci NS (2007). Conjugated polymer-based organic solar cells. Chem Rev.

[CIT0002] Scharber MC, Müjlbacher D, Koppe M (2006). Design rules for donors in bulk-heterojunction solar cells—towards 10% energy-conversion efficiency. Adv Mater.

[CIT0003] Facchetti A (2011). π-Conjugated polymers for organic electronics and photovoltaic cell applications. Chem Mater.

[CIT0004] Lu L, Zheng T, Wu Q (2015). Recent advances in bulk heterojunction polymer solar cells. Chem Rev.

[CIT0005] You J, Dou L, Hong Z (2013). Recent trends in polymer tandem solar cells research. Progr Polym Sci.

[CIT0006] Vohra V, Kawashima K, Kakara T (2015). Efficient inverted polymer solar cells employing favourable molecular orientation. Nat Photon.

[CIT0007] Liao S-H, Jhuo H-J, Yeh P-N (2014). Single junction inverted polymer solar cell reaching power conversion efficiency 10.31% by employing dual-doped zinc oxide nano-film as cathode interlayer. Sci Rep.

[CIT0008] Liu Y, Zhao J, Li Z (2014). Aggregation and morphology control enables multiple cases of high-efficiency polymer solar cells. Nat Comm.

[CIT0009] Liu C, Yi C, Wang K (2015). Single-junction polymer solar cells with over 10% efficiency by a novel two-dimensional donor-acceptor conjugated copolymer. ACS Appl Mater Interf.

[CIT0010] Chen J-D, Cui C, Li Y-Q (2015). Single-junction polymer solar cells exceeding 10% power conversion efficiency. Adv Mater.

[CIT0011] You J, Dou L, Yoshimura K (2012). A polymer tandem solar cell with 10.6% power conversion efficiency. Nat Comm.

[CIT0012] Chen C-C, Chang W-H, Yoshimura K (2014). An efficient triple-junction polymer solar cell having a power conversion efficiency exceeding 11%. Adv Mater.

[CIT0013] Brabec CJ (2014). Organic photovoltaics: technology and market. Sol Energ Mat Sol Cells.

[CIT0014] Balderrama VS, Avila-Herrera F, Guadalupe Sanchez J (2016). Organic solar cells toward the fabrication under air environment. IEEE J Photovolt.

[CIT0015] Norrman K, Madsnen MV, Gevorgyan SA (2010). Degradation patterns in water and oxygen of an inverted polymer solar cell. J Am Chem Soc.

[CIT0016] Schafferhans J, Baumann A, Deibel C (2008). Trap distribution and the impact of oxygen-induced traps on the charge transport in poly(3-hexylthiophene). Appl Phys Lett.

[CIT0017] Vohra V, Higashimine K, Tsuzaki S (2014). Formation of vertical concentration gradients in poly(3-hexylthiophene-2,5-diyl): phenyl-C61-butyric acid methyl ester-graded bilayer solar cells. Thin Solid Films.

[CIT0018] Jouane Y, Colis S, Shmerber G (2012). Annealing treatment for restoring and controlling the interface morphology of organic photovoltaic cells with interfacial sputtered ZnO films on P3HT:PCBM active layers. J Mater Chem.

[CIT0019] Liang C-W, Su W-F, Wang L (2009). Enhancing the photocurrent in poly(3-hexylthiophene)/[6,6]-phenyl C61 butyric acid methyl ester bulk heterojunction solar cells by using poly(3-hexylthiophene) as a buffer layer. Appl Phys Lett.

[CIT0020] Cho S, Kim K-D, Heo J (2014). Role of additional PCBM layer between ZnO and photoactive layers in inverted bulk heterojunction solar cells. Sci Rep.

[CIT0021] Lee KH, Schwenn PE, Smith ARG (2011). Morphology of all-solution-processed “bilayer” organic solar cells. Adv Mater.

[CIT0022] Ayzner AL, Tassone CJ, Tolbert SH (2009). Reappraising the need for bulk heterojunctions in polymer−fullerene photovoltaics: the role of carrier transport in all-solution-processed P3HT/PCBM bilayer solar cells. J Phys Chem C.

[CIT0023] Seok J, Shin TJ, Park S (2015). Efficient organic photovoltaics utilizing nanoscale heterojunctions in sequentially deposited polymer/fullerene bilayer. Sci Rep.

[CIT0024] Vohra V, Higashimine K, Murakami T (2012). Addition of regiorandom poly(3-hexylthiophene) to solution processed poly(3-hexylthiophene):[6,6]-phenyl-C61-butyric acid methyl ester graded bilayers to tune the vertical concentration gradient. Appl Phys Lett.

[CIT0025] Vohra V, Arrighetti G, Barba L (2012). Enhanced vertical concentration gradient in rubbed P3HT:PCBM graded bilayer solar cells. J Phys Chem Lett.

[CIT0026] Huang J-H, Ho Z-Y, Kuo T-H (2009). Fabrication of multilayer organic solar cells through a stamping technique. J Mater Chem.

[CIT0027] Kim JK, Kim W, Wang DH (2013). Layer-by-layer all-transfer-based organic solar cells. Langmuir.

[CIT0028] Wang DH, Choi D-G, Lee K-J (2010). Unexpected solid–solid intermixing in a bilayer of poly(3-hexylthiophene) and [6,6]-phenyl C61-butyric acidmethyl ester via stamping transfer. Org Electron.

[CIT0029] Tong J, Xiong S, Li Z (2015). Vacuum-free and metal electrode-free organic tandem solar cells. Appl Phys Lett.

[CIT0030] Jiang F, Liu T, Zeng S (2015). Metal electrode–free perovskite solar cells with transfer-laminated conducting polymer electrode. Opt Expr.

[CIT0031] Carlson A, Bowen AM, Huang Y (2012). Transfer printing techniques for materials assembly and micro/nanodevice fabrication. Adv Mater.

[CIT0032] Juang F-S, Chittawanij A, Hong L-A (2015). The study of N-type doping and stamping transfer processes of electron transport layer for organic light-emitting diodes. IEICE Trans Electron.

[CIT0033] Stalder AF, Kulik G, Sage D (2006). A snake-based approach to accurate determination of both contact points and contact angles. Colloids Surf A.

[CIT0034] Porzio W, Scavia G, Barba L (2014). On the packing and the orientation of P(NDI2OD-T2) at low molecular weight. Eur Pol J.

[CIT0035] Berg JC (2012). An introduction to interfaces & colloids: the bridge to nanoscience.

[CIT0036] Dupré A, Dupré P (1869). Théorie mécanique de la chaleur.

[CIT0037] Razali NT, Osaka I, Takimiya K (2014). H. Achieving high efficiency and stability in inverted organic solar cells fabricated by laminated gold leaf as top electrodes. Appl Phys Expr.

[CIT0038] Owen MJ (1998). First international congress on adhesion science and technology.

[CIT0039] Petrosino M, Rubino A (2012). The effect of the PEDOT:PSS surface energy on the interface potential barrier. Synt Met.

[CIT0040] Wilken S, Hoffmann T, von Hauff E (2012). ITO-free inverted polymer/fullerene solar cells: interface effects and comparison of different semi-transparent front contacts. Sol Energ Mat Sol Cells.

[CIT0041] Torchinsky I, Rosenman G (2009). Wettability modification of nanomaterials by low-energy electron flux. Nanoscale Res Lett.

[CIT0042] Tajima Y, Matsuura T, Numata Y (2008). Surface free energy and wettability determination of various fullerene derivative films on amorphous carbon wafer. Jpn J Appl Phys.

[CIT0043] Lim FJ, Ananthanarayanan K, Luther J (2012). Influence of a novel fluorosurfactant modified PEDOT:PSS hole transport layer on the performance of inverted organic solar cells. J Mater Chem.

[CIT0044] Kuwabara T, Omura Y, Yamaguchi T (2014). Factors affecting the performance of bifacial inverted polymer solar cells with a thick photoactive layer. J Phys Chem C.

[CIT0045] Xue B, Vaughan B, Poh C-H (2010). Vertical stratification and interfacial structure in P3HT:PCBM organic solar cells. J Phys Chem C.

[CIT0046] Kuwabara T, Nakashima T, Yamaguchi T (2012). Flexible inverted polymer solar cells on polyethylene terephthalate substrate containing zinc oxide electron-collection-layer prepared by novel sol–gel method and low-temperature treatments. Org Electron.

[CIT0047] Zimmermann B, Würfel U, Niggemann M (2009). Longterm stability of efficient inverted P3HT:PCBM solar cells. Sol Energ Mat Sol Cells.

